# Surgical Fixation of Bilateral Simultaneous Avulsion Fractures of the Proximal Tibia in a 12-Year-Old with History of Conservatively Managed Unilateral Tibial Avulsion Fracture

**DOI:** 10.1155/2017/5925421

**Published:** 2017-04-03

**Authors:** Christopher Newman, Dharsh Musiienko, Samuel Law

**Affiliations:** Western Health Department of Orthopaedics, Footscray Hospital, Gordon Street, Footscray, VIC 3011, Australia

## Abstract

Fractures of the proximal tibial epiphysis are rare, representing less than 3% of all epiphyseal and 1% of all physeal injuries in adolescents. Bilateral injuries are extremely rare. The specific anatomical and histological features of the proximal tibial epiphysis make it vulnerable to a specific fracture pattern that occurs when the tensile force of the quadriceps is greater than the fibrocartilaginous tissue underlying the tibial tuberosity. We report the first case to our knowledge of a 12-year-old boy who sustained simultaneous bilateral tibial avulsion fractures on the background of a previous conservatively managed unilateral tibial tuberosity avulsion fracture. We report this case for its uniqueness and as an educational review of the anatomy, the mechanism of injury, and the development of classifying these fractures and discussion of the stages of the growing physis that determine the treatment approach.

## 1. Introduction

**Figure 1:**
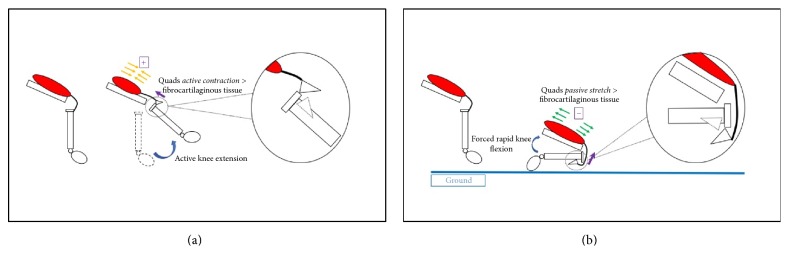
(a) Schematic representation of quadriceps contraction causing proximal tibial avulsion fracture. (b) Schematic representation of forced quadriceps stretch causing proximal tibial avulsion fracture.

Fractures of the tibial tuberosity were first described in the early 1950s. They are considered rare, representing less than 3% of all epiphyseal and 1% of all physeal injuries in adolescents [1–4]. Bilateral injuries are extremely rare, with 21 cases described in the literature [5–25]. The first case of simultaneous bilateral avulsion fractures of the tibial tubercle was reported by Borch-Madsen [23] in 1954. These fractures occur at the anterior tibial ossification centre. Importantly, the epiphysiodesis progresses from the posterior to the anterior part of the tibia. Therefore, the anterior part of the proximal tibia persists as a histologically weaker region than the posterior part of the proximal tibia secondary to the cell type in that region. Underlying the tibial tuberosity in the growing adolescent is fibrocartilaginous tissue that is gradually being replaced in a proximal to distal direction by columnar cartilage cells. These columnar cells are structurally weak and, therefore, cannot withstand the large tensile forces exerted by the knee extensor mechanism, especially where the quadriceps insert at the weaker anterior aspect. This explains the occurrence of avulsion fractures in adolescents in this region. The epiphyseal plate fuses completely between the ages of 13 and 15 years in girls and the ages of 15 and 19 years in boys [24], meaning that athletic young males with powerful quadriceps tendons relative to their bone maturity are at an increased risk of avulsion fractures. Different mechanisms have been proposed by various authors [24, 25]; however in simple terms the avulsion occurs when the tensile force of the quadriceps is greater than the fibrocartilaginous tissue underlying the tibial tuberosity. This happens usually during quadriceps muscle contraction (Figure 1(a)) or where the knee is rapidly forced into flexion. In the latter situation, the adolescent strikes the knee(s) or tibia on a surface such as the ground, and the weight of their body forces the knee into flexion (Figure 1(b)).

**Figure 2:**
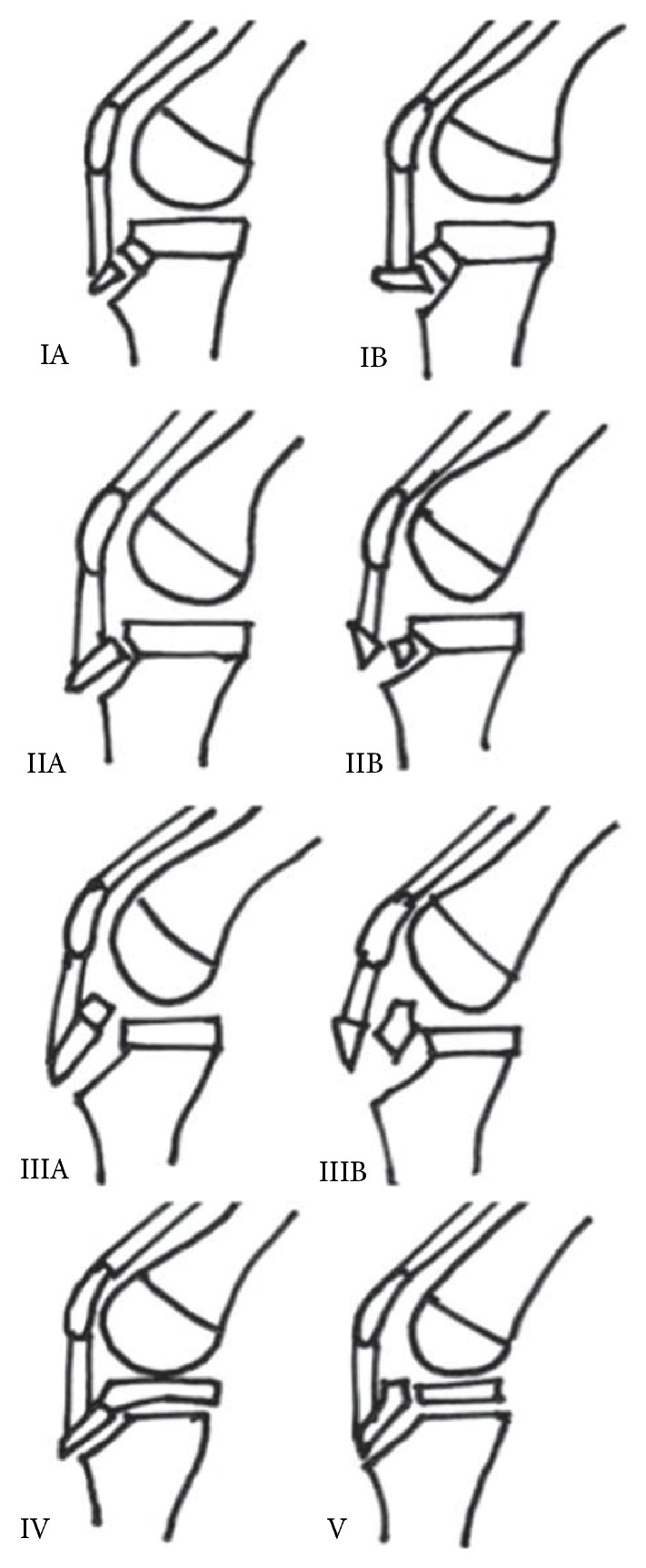
Classification of proximal tibial tuberosity fractures in adolescents [adapted from Ryu and McKoy].

Several classification systems have been described to categorise these fractures, which can be confusing to the reader given the use of subcategories. With respect to classifying these injuries it is important to be familiar with the anatomy of the growing proximal tibia and that it has two ossification centres: (i) the primary ossification centre (proximal) and (ii) the secondary ossification centre (distal at the distal aspect of the tibial tuberosity). The Watson-Jones classification used the fracture location relative to the ossification centres with types I, II, and III being distal to the ossification centre, through the ossification centres and extending proximally through the joint, respectively [26]. Ogden et al. [27] subdivided this classification into A (noncomminuted fractures) and B (comminuted fractures). Later, Ryu and Debenham [28] added a type IV fracture, defined as a fracture of the tibial epiphysis with posterior extension. Most recently McKoy and Stanitski [29] suggested the addition of a type V two-part fracture, which is a “Y” shaped fracture pattern in the proximal region of the knee to complete the classification system (Figure 2). These systems in the knee are often preferred to the traditional Salter-Harris classification because of the duel ossification centres; however some overlap does apply.

## 2. Case Presentation

A 12-year-old Vietnamese boy presented to our emergency department with bilateral knee pain and swelling. The mechanism of injury was sustained as a result of tripping with bilateral knee strikes on the ground while playing sports at school. The subject and his parents denied jumping in relation to the injury or any knee pain prior to the injury. The history revealed immediate pain and the inability to mobilise or bear weight.

On physical examination, the boy had an athletic constitution. There was significant appreciable bilateral knee swelling with a fixed flexion deformity on observation with pain being the limiting factor disabling knee extension. Generalised tenderness and an intra-articular effusion were present on palpation with the predominant source of pain at the tibial tuberosities. Any attempt of motion provoked severe pain. Given the pain, it was difficult to assess the integrity of the cruciate and collateral ligaments; however the limited examination did not reveal obvious instability. Neurovascular examination was normal and compartments were soft.

Interestingly, the boy's past history was significant for a type IIIA left knee avulsion fracture that he sustained 13 months priorly. This happened via a different mechanism (jumping) where pain was felt while pushing off. On that occasion an MRI showed an injury to both ends of the patella ligament with some avulsion of the patella proximally and some lifting up of the tibial tuberosity distally. He was managed conservatively on that occasion in a hinged-knee brace locked in extension for six weeks with physiotherapy to progress ROM thereafter. The paediatric orthopaedic surgeon who reviewed him in the outpatient clinic made a specific note in his letter stating that “this injury will have changed the relative length of his patella by a small amount”, going on to say that “we will need to take this slowly in terms of protecting this.”

**Figure 3:**
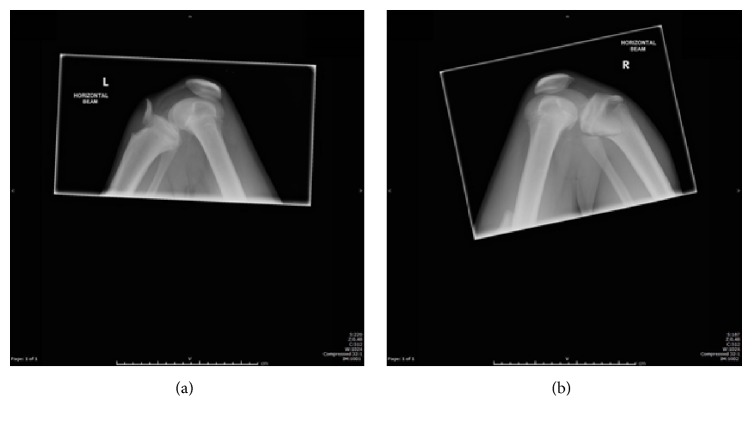
Plain radiographs of left (a) and right (b) proximal tibial avulsion fractures, respectively.

**Figure 4:**
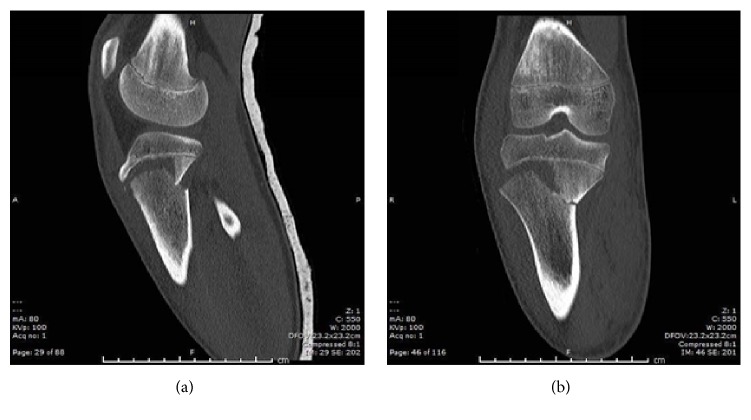
Sagittal (a) and coronal (b) slices of noncontrast CT scan of patient's right knee demonstrating grade IV proximal tibial avulsion fracture.

On this presentation, plain radiographs (Figures 3(a) and 3(b)) showed bilateral tibial tuberosity avulsion fractures. The previously injured left knee was a grade IIIB injury. The right knee was a grade IV injury in the Ryu and Debenham classification or a Salter-Harris II injury. A CT of the right knee confirmed this (Figure 4).

**Figure 5:**
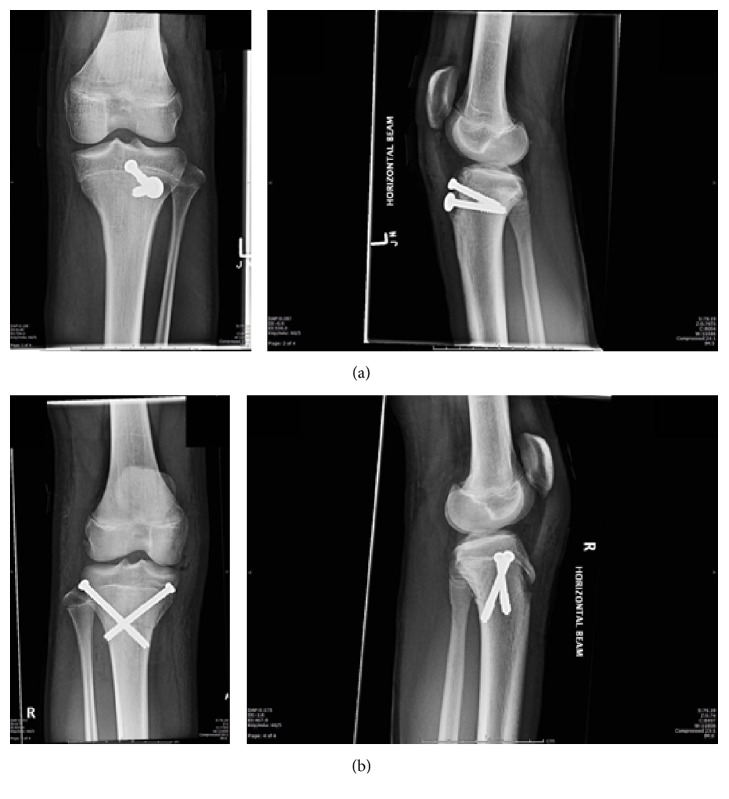
Day 1 postoperative plain radiographs of left (a) and right (b) knees following ORIF.

Sequential bilateral open reduction and internal fixation (ORIF) was undertaken within 12 hours of injury. Fixation was achieved under radiological guidance. Both knees were fixed using 2 × 6.5 mm cannulated screws, with the right knee having a crossed configuration (Figures 5(a) and 5(b)). Intraoperative assessment of the left knee suggested a refracturing of the previous injury sustained 13 months priorly. Postoperatively, the boy was placed in bilateral Zimmer knee splints for 6 weeks. He was restricted to non-weight-bearing during this time. On assessment 6 weeks postoperatively, he was transitioned to a hinged-knee brace bilaterally, which was unlocked on the right and locked from 0 to 30 degrees of extension on the left and he was allowed protected weight-bearing. 10 weeks postoperatively he had significant quads wasting and had 5 degrees of extension lag on the left knee but he was able to bear weight unaided and without braces. His follow-up radiographs showed ongoing healing of the fractures.

## 3. Discussion

Proximal tibial physeal injuries are rare, and it is thought that this is due to an inherent stability afforded by the anatomical arrangement of the epiphysis, especially with respect to varus and valgus forces. These coronal forces bypass the epiphysis because structures such as the medial collateral ligament medially and the fibula laterally transmit these forces to the metaphysis. Sagittal forces, however, which can be from either repetitive microtrauma or acute fractures from high-energy tensile force from the quadriceps, can cause significant epiphyseal trauma. These fractures have been suggested to be associated with some predisposing conditions and diseases, such as osteogenesis imperfecta [17], Osgood-Schlatter disease [10], and more recently vitamin D deficiency [30].

This case serves to illustrate a number of points with respect to proximal tibial physeal injuries. To the best of our knowledge this case is the first of its kind where the patient sustained bilateral tibial avulsion injuries that underwent operative fixation on the background of a conservatively managed unilateral avulsion injury just over a year priorly. It also not only demonstrates the two proposed mechanisms (muscle contraction or rapid forced knee flexion) (Figures 1(a) and 1(b)) for the injury to occur at different points in time for the same patient, but also serves to educate on the growth of classification systems with respect to this injury. As stated in the case, patient records revealed a letter from the attending outpatient orthopaedic surgeon expressing his thoughts about this boy needing a slow recovery with appropriate physiotherapy following his first injury. It is not known whether this boy adequately participated in rehabilitation sessions; however it is likely that his initial injury predisposed him to reinjuring his left knee, reenforcing the importance of taking the rehabilitation slow following conservative management of these injuries. Furthermore, the left knee sustained a different type of fracture than the right, which also could be considered secondary to his prior injury. Finally, it is also interesting to note that in the same outpatient letter from the attending orthopaedic surgeon suggested that the relative length of his patella ligament may have changed by a small amount following the boy's initial injury. It could be argued therefore that this change in length altered the sagittal force directed on the previously injured knee.

Treatment of these fractures is dependent on various factors, including the degree of displacement of the fragments and the level of skeletal maturity of the patient. Since the partially closed epiphysis is an indicator for high skeletal maturity, limb length discrepancies and angular deformities are not expected posttraumatically [31]. Omar et al. [32] describe these injuries as “transitional fractures,” which should be differentiated from true Salter-Harris fractures because the tibia is reaching skeletal maturity. It has been suggested that injuries that are either type I or type II or are undisplaced can be treated conservatively. Injuries that are classified as types III, IV, or V are usually fixed surgically. We used 6.5 mm cannulated screws in our patient; however plates and screws [32] and tension band wiring [33] have also been used. The cannulated screw placement for each knee in our patient was determined by the fracture pattern using a lag technique, which aims to achieve screw placement as close to perpendicular to the fracture line as possible. Given this 12-year-old boy is approaching physeal closure (as demonstrated by lateral projections in Figure 2), damage to the physes was not of great concern. The primary aim was to achieve adequate fracture reduction. With hindsight, given the initial injury was a type IIIA injury, albeit an undisplaced injury, it could be argued that surgical fixation in the first instance may have prevented subsequent injury in the original knee, especially given that excellent surgical outcomes have been reported regardless of fixation method. Metalware removal is not required following this operation; however counselling the parents regarding possible irritation of the skin while kneeling or of the quadriceps muscle at terminal extension and during exercise is important. Should this occur, the screws can be removed at a later date.
